# A Prediction Algorithm for Drug Response in Patients with Mesial Temporal Lobe Epilepsy Based on Clinical and Genetic Information

**DOI:** 10.1371/journal.pone.0169214

**Published:** 2017-01-04

**Authors:** Mariana S. Silva-Alves, Rodrigo Secolin, Benilton S. Carvalho, Clarissa L. Yasuda, Elizabeth Bilevicius, Marina K. M. Alvim, Renato O. Santos, Claudia V. Maurer-Morelli, Fernando Cendes, Iscia Lopes-Cendes

**Affiliations:** 1 Department of Medical Genetics, University of Campinas—UNICAMP, and the Brazilian Institute of Neuroscience and Neurotechnology (BRAINN), Campinas, São Paulo, Brazil; 2 Department of Statistics, Institute of Mathematics, Statistics and Scientific Computing, University of Campinas—UNICAMP, and the Brazilian Institute of Neuroscience and Neurotechnology (BRAINN), Campinas, São Paulo, Brazil; 3 Department of Neurology, University of Campinas—UNICAMP, and the Brazilian Institute of Neuroscience and Neurotechnology (BRAINN), Campinas, São Paulo, Brazil; Cleveland Clinic, UNITED STATES

## Abstract

Mesial temporal lobe epilepsy is the most common form of adult epilepsy in surgical series. Currently, the only characteristic used to predict poor response to clinical treatment in this syndrome is the presence of hippocampal sclerosis. Single nucleotide polymorphisms (SNPs) located in genes encoding drug transporter and metabolism proteins could influence response to therapy. Therefore, we aimed to evaluate whether combining information from clinical variables as well as SNPs in candidate genes could improve the accuracy of predicting response to drug therapy in patients with mesial temporal lobe epilepsy. For this, we divided 237 patients into two groups: 75 responsive and 162 refractory to antiepileptic drug therapy. We genotyped 119 SNPs in *ABCB1*, *ABCC2*, *CYP1A1*, *CYP1A2*, *CYP1B1*, *CYP2C9*, *CYP2C19*, *CYP2D6*, *CYP2E1*, *CYP3A4*, and *CYP3A5* genes. We used 98 additional SNPs to evaluate population stratification. We assessed a first scenario using only clinical variables and a second one including SNP information. The random forests algorithm combined with leave-one-out cross-validation was used to identify the best predictive model in each scenario and compared their accuracies using the area under the curve statistic. Additionally, we built a variable importance plot to present the set of most relevant predictors on the best model. The selected best model included the presence of hippocampal sclerosis and 56 SNPs. Furthermore, including SNPs in the model improved accuracy from 0.4568 to 0.8177. Our findings suggest that adding genetic information provided by SNPs, located on drug transport and metabolism genes, can improve the accuracy for predicting which patients with mesial temporal lobe epilepsy are likely to be refractory to drug treatment, making it possible to identify patients who may benefit from epilepsy surgery sooner.

## Introduction

Mesial temporal lobe epilepsy (MTLE) is the most common form of epilepsy in adults, and it is frequently associated with hippocampal sclerosis (HS), which can be detected by magnetic resonance imaging (MRI) [1, 2]. A high proportion of patients with MTLE is refractory to drug treatment with antiepileptic drugs (AEDs), and the presence of HS has been used to predict poor AED response in these patients [1]. However, other factors have been implicated in AED drug response (yet not proven), such as gender, age of seizure onset, aetiology of epilepsy, as well as genetic factors including family history of epilepsy and gene mutations affecting proteins involved in AED absorption, metabolism, and transport [3].

Some AEDs, such as phenytoin (PHT) and carbamazepine (CBZ), are substrates for adenosine triphosphate-binding cassette transporter proteins (ABC) [4, 5]. *ABC* genes encode multidrug resistance proteins, and previous studies have suggested that multidrug resistance proteins could decrease drug penetration into the brain of patients with refractory MTLE [4, 6], leading to pharmacoresistance. In fact, studies showed an increase in ABCB1 and ABCC2 protein expression in endothelial cells from temporal lobe blood vessels of patients with refractory MTLE [6, 7], and a higher ABCB1 protein activity was observed in hippocampus of patients with pharmacoresistant temporal lobe epilepsy [8]. In addition, because PHT and CBZ are subjected to hepatic metabolism, cytochrome P450 (CYP450) isoenzymes could have an important role, affecting drug exposure in brain tissue, these include CYP2C9, CYP2C19, CYP2D6, CYP3A4, CYP3A5, CYP1B1 and CYP2E1 proteins [9–13].

Genetic studies have demonstrated that single nucleotide polymorphisms (SNPs) located in the *ABCB1* (rs1045642) and *ABCC2* genes (rs2273697, rs717620 and rs3740066) could be associated with refractory epilepsy [14, 15]. However, subsequent studies did not replicate these findings [16, 17]. Similarly, SNPs in *CYP2C9* (rs1799853 and rs1057910) have been associated with PHT dose requirement [18, 19], and SNPs in *CYP2C19* (rs12248560) and *CYP2D6* have been considered pharmacogenomic biomarkers for neurologic and psychiatric therapeutic drugs, including clobazam (CLB) and diazepam, receiving approval for use by the U.S. Food and Drug Administration (FDA) [19]. In addition, studies have showed association between rs2606345 in *CYP1A1* gene and North Indian women with epilepsy [20], and between rs762551 in *CYP1A2* and CBZ pharmacokinetics in children diagnosed with partial or generalized tonic-clonic seizures [21]. However, none of these pharmacogenetic variables have been proven to be useful for predicting response to AEDs in patients with MTLE [22]. In this context, the objective of this study was to evaluate whether SNPs in the following genes—*ABCB1*, *ABCC2*, *CYP1A1*, *CYP1A2*, *CYP1B1*, *CYP2C9*, *CYP2C19*, *CYP2D6*, *CYP2E1*, *CYP3A4*, and *CYP3A5—*could improve the accuracy of predicting poor response to AED therapy in patients with MTLE.

## Methods

### Subjects and clinical evaluation

The Research Ethics Committee of our institution (Comitê de Ética em Pesquisa da Universidade Estadual de Campinas–UNICAMP) approved this study and all 237 participants signed a consent form before entering the study. We assessed consecutive patients with MTLE (103 males) classified according to the International League Against Epilepsy (ILAE) criteria [23]. We followed all patients regularly from 2006 to 2015 at the outpatient epilepsy clinic of the University of Campinas-(UNICAMP) hospital, which is a tertiary centre for epilepsy, and in a community outpatient clinic in Campinas, Brazil. Two investigators (MSS and EB) interviewed all patients and collected information using structured questionnaire gathering information regarding age and frequency of seizures at epilepsy onset, febrile seizures (FS), presence of initial precipitating injuries (IPIs), and number of AEDs. In addition, all patients underwent a neurological exam, serial interictal EEGs with a duration of at least 35 minutes, and high resolution MRI epilepsy protocol, including thin (3mm or less) T1 and T2 sequences in the axial, coronal and sagittal planes as well as a 3D T1-weighted acquisition with 1mm isotropic voxels in a 2T (Elscint Prestige, Haifa, Israel) or 3T (Philips Intera Achieva, Best, the Netherlands) scanner [24]. All data were reviewed by a senior epileptologist (FC).

MTLE was defined based on a constellation of signs and symptoms, with the characteristic seizure semiology, including viscerosensory or experiential auras, such as rising epigastric sensation, fear, déjà vu, jamais vu, dreamy state, with or without autonomic symptoms (e.g. flushing, pallor, tachycardia), frequently followed by dyscognitive seizures with staring, oral, verbal, or gestural automatisms (e.g., lip smacking, chewing) with a progressive clouding of consciousness, sometimes with dystonic posture of the contralateral hand [23, 25]. Interictal EEG studies presented either lateralised anterior or middle temporal epileptiform activity or no epileptiform abnormalities and normal background. Patients with extratemporal epileptiform or non-epileptiform abnormalities were not selected. Video-EEG for seizure recording, neuropsychological tests and positron emission tomography (PET) or ictal single photon emission computed tomography (SPECT) were performed in a subset of patients; in particular, those with refractory seizures, unclear interictal EEGs and negative MRIs.

Hippocampal atrophy and other MRI signs of HS were assessed by visual analyses by two epilepsy imaging experts (CLY and FC) and the images were classified as having normal findings or signs of HS. Classic signs of HS were as follows: reduction of volume and/or abnormal shape observed on T1-weighted images, with or without increased signal observed in T2-weighted and FLAIR images [25]. Special attention was given to rule out subtle signs of other lesions, such as focal cortical dysplasia, tumours or gliotic scars. Patients with dual pathology or other MRI abnormalities were not included. In addition, hippocampal volumetry was performed in all patients and compared to a group of 79 healthy controls (with similar age and sex distribution) with FreeSurfer software v.5.1.0 (http://surfer.nmr.mgh.harvard.edu), as previously described [24]. Therefore, the presence of MRI findings of HS or normal MRI was defined by results of visual analyses and automatic hippocampal volumetry.

We divided patients into two groups: 75 (32%) individuals were classified as AED responsive MTLE and 162 (68%) were classified as refractory MTLE. AED responsive was defined as freedom from all seizures, including auras, for a period of 12 months or at least three times the previous longest seizure-free interval, whichever was longer, according to the recent definition of the ILAE [26]. Refractory MTLE included patients who failed to achieve sustained seizure freedom, after two or more trials of tolerated and appropriately chosen AED schedules in monotherapies or in combination [26].

### SNP selection

In order to represent variability across candidate genes, we perform a systematically SNPtag selection using SNPBROWSER^TM^ v.4.0 software (Applied Biosystems, Foster City, California, USA), based on the International HapMap Project (http://hapmap.ncbi.nlm.nih.gov/), a minimum allele frequency (MAF)>0.05, and a pairwise linkage disequilibrium (LD) of r^2^>0.8. In this case, we did not include intentionally the SNPs previously reported because it could lead to a bias in SNPtag candidate gene screening. Even so, the systematic selection led to the inclusion of four previously reported ones (rs1045642; rs2273697; rs3740066; rs1057910), among 119 selected SNPs for the analysis.

**Fig 1:**
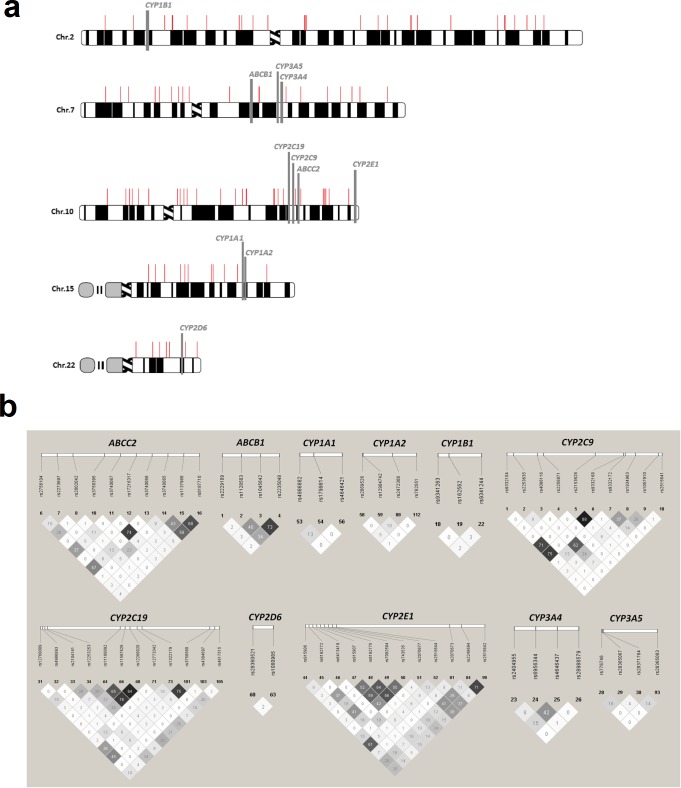
Chromosome location of candidate genes and SNPtag map. a) genes are indicated by the grey vertical bars and distribution of SNPs used in the population structure are indicated by the red vertical bars; b) linkage disequilibrium estimates are presented in terms of pairwise r^2^ values. Values of r^2^ > 80 indicate linkage disequilibrium.

Furthermore, we used an additional 98 SNPs for population stratification analysis, since 98 slots were available from 48-Plex kits in our laboratory, and previous studies showed that any number of SNPs greater than 65 is adequate to evaluate population stratification [27]. However, since the human genome contains a high density of SNPs, we developed a bioinformatics pipeline to select randomly SNPs for the population stratification analysis. Briefly, using the International HapMap Project, a SNP frequency vector was built based on the following parameters: MAF>0.05, and minimum distance between two SNPs = 300 kb, which avoid possible LD [28]. In the next step, the pipeline uses the ‘sample’ function in R software v.3.2.3 (http://www.r-project.org) to select randomly 98 SNPs from the SNP frequency vector. We concentrated SNP selection on chromosomes 2, 7, 10, 15 and 22 (Fig 1A), which harbour the candidate gene analysed, since previous studies showed that population structure could vary among chromosomes. These differences could be due to the presence of natural selection imprint observed in many regions across the genome, thus creating substantial variation and affecting the degree of variance inflation for specific loci, which leads to biased analyses [29].

### SNPs genotyping

We obtained genomic DNA from patients peripheral blood using standard procedure [30], and quantified using NanoVue V1.7.2 Spectrophotometer (GE Healthcare, Chicago, Illinois, USA). In order to genotype the SNPs, we used the SNPlex^TM^ Genotyping System 48-plex technology and ABI3730xl equipment (Applied Biosystems, Foster City, CA, USA). We used the GeneMapper v.4.0 Software (Applied Biosystems, Foster City, CA, USA) to identify the SNP genotypes from fluorescence signals.

### *ABCC2* mRNA quantification

We obtained total RNA from hippocampal tissue that had been surgically removed from 11 patients with refractory MTLE who underwent epilepsy surgery and had histopathology confirming HS. Among them, nine were also included in the SNP genotyping study. All 11 patients had pre-surgical MRIs with sings of hippocampal sclerosis. In addition, we obtained normal hippocampal tissue from six autopsies of patients who died of non-neurological-related disorders. We extracted total RNA by Trizol^®^ protocol (Life Technologies, Carlsbad, CA, USA), and assessed RNA quality by NanoVue spectrophotometer at 260/280 nm (GE Healthcare, Buckinghamshire, UK). We quantified the transcripts in triplicate using RT-PCR TaqMan^®^ System and ABI 7500 equipment (Applied Biosystems, Foster City, CA, USA). We used a target *ABCC2* assay (probe ID: Hs00166123_m1) and human 18S rRNA as an endogenous control. We calculated the relative quantification using fluorescence signals by the comparative threshold cycle method [31].

### Statistical and prediction analysis

We used R software to perform all statistical analyses. We compared categorical clinical variables (gender, febrile seizures, and presence of HS on MRI) using Fisher’s exact test, and Students’ two sample t-test to compare mean age at onset of seizures between the two groups. We evaluated the genotyped SNPs in terms of Hardy–Weinberg equilibrium (p-value > 0.01), MAF>0.01 and pairwise LD (r^2^>0.8) by HAPLOVIEW v.4.2 software [32]. In order to evaluate whether response and refractory population substructure is similar, we performed the Analysis of Molecular Variance (AMOVA) by ARLEQUIN v3.5.1.2 software [33], using the 98 additional SNPs as factors in the analysis. The AMOVA partitions the source of genetic variance into two components: within-groups and between-groups, and measures the population differentiation due to genetic structure by the fixation index (Fst). We considered Fst<0.05 as the absence of population stratification [29], as well as the between-groups variance component < 0.7%, because this value was the smallest one found when comparing populations within regions worldwide [34].

We coded the SNP genotypes as AA, AB and BB, where B represents the alternative allele. To evaluate whether SNPs improve the accuracy of predicting refractory MTLE, we used three different scenarios: one that takes into account only clinical variables, one including only SNP genotypes, and a third that includes clinical variables and SNP genotypes combined.

We used the random forests approach to select the best predictive model [35], which is able to handle big data with a large number of variables (SNPs and clinical data), but limited sample size [35, 36]. Random forests have one parameter *m*_*try*_ that describes the number of randomly selected variables used to build the classification trees. To estimate the best mixture of parameters *m*_*try*_ for each of 5000 classification trees, we used the Leave-One-Out Cross-Validation (LOOCV) [35]. This validation divides the sample in one group containing only one individual (called test set) and another group containing the remained individuals (called training set). The algorithm fits the model from the training set and predicts the test set based on the training set, repeating this step until all individuals were tested (*n* steps, where *n* = sample size). In the end, we selected the best model identifying the *m*_*try*_ that maximises the area under the curve (AUC) among the 5000 classification trees. In addition, each variable had its importance rank within the model estimated in terms of mean decreased accuracy (MDA). The MDA calculates the discriminatory accuracy using a set of predictor variables. Then, the algorithm permutes (or excludes) one variable *X* from the variable set and calculates a second discriminatory accuracy. We estimate MDA by the difference between the accuracy before and after permuting *X*. In this case, if the variable is important for the model, the discriminatory accuracy decreases substantially, and the greater the accuracy of the random forest decreases due to the permutation (or exclusion) of a single variable, the more important it is considered [35].

## Results

### Clinical evaluation

As shown in Table 1, 61.3% of responsive and 54.3% of refractory MTLE patients were female. The mean age of seizure onset was 12.4 years old for responsive and 11.3 years old for refractory patients. In addition, most patients presented the first seizure before 20 years old. Among the clinical variables, only the distribution of HS was different between patients with responsive and refractory MTLE (Fisher’s exact test p-value<6.43e^-06^). Among patients, four did not have any information regarding previous history of febrile seizure, and one could not recall age at seizure onset; therefore, they were withdrawn from the prediction analysis. We used the age of epilepsy onset as a categorical variable, classifying individuals in 10 groups based on five year windows.

**Table 1:** Descriptive statistics of the two groups of patients with MTLE.

Variable		AED Responsive (n = 75)	AED Refractory (n = 162)	p–value
**Gender**	Male	29 (38.7%)	74 (45.7%)	0.39
	Female	46 (61.3%)	88 (54.3%)	
**Mean age of seizure onset (years)**		12.4 (SD = 10.8)	11.3 (SD = 8.5)	0.52
**Age of seizure onset distributed by groups (range in years)**	1 (0–5)	23 (30.7%)	52 (32.1%)	0.18
	2 (6–10)	12 (16.0%)	30 (18.6%)	
	3 (11–15)	17 (22.7%)	35 (21.6%)	
	4 (16–20)	10 (13.3%)	22 (13.6%)	
	5 (21–25)	3 (4.0%)	13 (8.0%)	
	6 (26–30)	5 (6.7%)	6 (3.7%)	
	7 (31–35)	2 (2.7%)	1 (0.6%)	
	8 (36–40)	1 (1.3%)	1 (0.6%)	
	9 (41–45)	1 (1.3%)	1 (0.6%)	
	10 (> 46)	1 (1.3%)	0 (0.0%)	
	Unknown	0 (0.0%)	1 (0.6%)	
**Febrile seizures**	Yes	16 (21.3%)	35 (21.6%)	1.00
	No	58 (77.3%)	124 (76.5%)	
	Unknown	1 (1.4%)	3 (1.9%)	
**Hippocampal sclerosis**	Yes	50 (66.7%)	148 (91.4%)	6.43e^-06^
	No	25 (33.3%)	14 (8.6%)	

AED, antiepileptic drug; SD, standard deviation.

In addition, CBZ, PHT and CLB appeared as the main AED used in mono or polytherapy among responsive and refractory patients. Notably, diazepam, nitrazepam, levetiracetam, and lacosamide were less commonly used (Table 2). AED treatment information was acquired based on pre-operative data for refractory patients who underwent surgery, and the sum of percentages of polytherapy groups is higher than 100% because one individual could use more than one drug. We provided detailed information for each patient in S1 and S2 Tables.

**Table 2:** Percentage that each AED appears in monotherapy or polytherapy in the responsive and refractory patients.

AED	Monotherapy		Polytherapy	
	Responsive (total = 30)	Refractory (total = 29)	Responsive (total = 45)	Refractory (total = 133)
Carbamazepine	70.0%	100.0%	62.2%	81.2%
Phenitoin	13.3%	0.0%	28.9%	9.0%
Phenobarbital	16.7%	0.0%	22.2%	4.5%
Clobazam	0.0%	0.0%	44.4%	64.7%
Valproic acid	0.0%	0.0%	4.4%	8.3%
Topiramate	0.0%	0.0%	6.7%	7.5%
Lamotrigine	0.0%	0.0%	8.9%	11.3%
Oxcarbazepine	0.0%	0.0%	0.0%	3.8%
Clonazepam	0.0%	0.0%	2.2%	3.0%
Diazepam	0.0%	0.0%	2.2%	0.7%
Nitrazepam	0.0%	0.0%	0.0%	0.7%
Levetiracetam	0.0%	0.0%	0.0%	0.7%
Lacosamide	0.0%	0.0%	0.0%	0.7%

AED, antiepileptic drug.

### SNPs genotyping

The SNP average genotypes call rate was 91.7% (standard deviation = 4.7%) in the candidate genes and 84.0% (standard deviation = 12.2%) for SNPs used in the population stratification analysis. Among the 119 SNPs genotyped in the candidate genes, 23 were not in Hardy-Weinberg equilibrium or presented MAF<0.01. Additionally, 30 had genotype call rate<90.0% (S3 Table). Therefore, we excluded these 53 SNPs from further analysis, remaining 38 patients with responsive MTLE and 84 patients with refractory MTLE who presented 100% genotype call rate and were suitable for prediction analysis (S1–S5 Tables). In addition, LD was observed between rs2153628 and rs9332168 (r^2^ = 0.88) in the *CYP2C9* gene, and between rs11597626 and rs12268020 (r^2 =^ 0.84) in *CYP2C19* in the remaining SNP set (Fig 1B). Among the 98 SNPs selected for population stratification analysis, 20 were not in Hardy-Weinberg equilibrium or presented an MAF<0.01, and 65 had a genotype call rate<90.0% (S4 Table). However, we only excluded 20 SNPs of the population stratification analysis, since the locus-by-locus AMOVA is robust for dealing with missing data [33]. As shown in Table 3, we observed Fst<0.05 for both chromosomes combined and separated. In addition, population stratification analysis estimated between-groups variance component of 0.3% for all five of the chromosomes combined, indicating that the two groups did not present differences in genetic structure with each other. Genotypes for all individuals included in this study are available in the supporting material accompanying this paper and will be deposited in a public genomic database (www.bipmed.org).

**Table 3:** Fst values from the sample for all five chromosomes combined and separated. The table also includes the percentage of genetic variation between and within groups.

Chromosome	Source of variation (%)		Fst
	Within-groups	Between-groups	
**Combined**	99.7	0.3	0.003
**2**	100.0	0.0	0.000
**7**	100.0	0.0	0.000
**10**	99.8	0.2	0.002
**15**	99.0	1.0	0.010
**22**	97.5	2.5	0.025

### Prediction analysis

**Fig 2:**
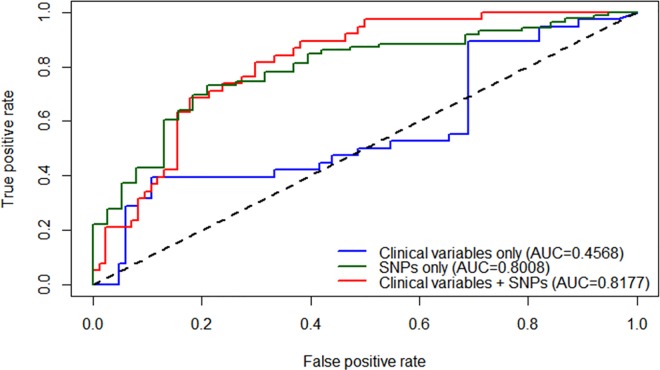
ROC curve showing the true positive rate (sensitivity), in function of false positive rate (1-specificity). The blue line indicates the prediction scenario using only clinical variables (hippocampal sclerosis, age of onset epilepsy, febrile seizures, and gender). The red line indicates the second scenario using the clinical variables plus SNPs. The dark green line indicates the scenario using only SNP genotypes. The area under the curve (AUC) values is showed for the three scenarios. The diagonal dashed line indicates a non-informative prediction (AUC = 0.5).

**Fig 3:**
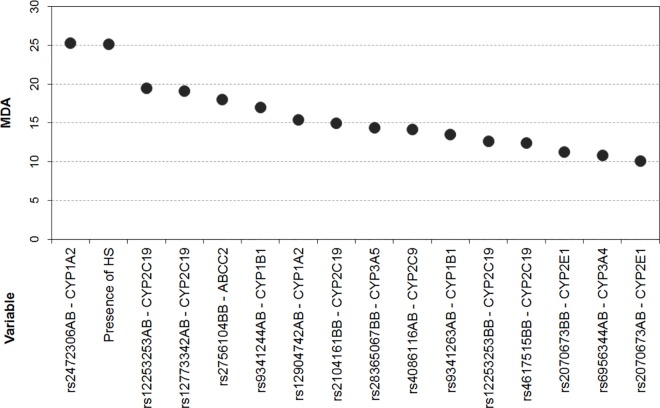
Variable importance plot. Each point represents the mean decreased accuracy estimate (y-axis), for each clinical variable and SNP genotype selected by the model (x-axis). Letter codes (AB and BB), after SNP name, indicate heterozygote and the alternative homozygote allele for each SNP, respectively.

The accuracy for identify a patient with refractory MTLE using only clinical information was 0.4568 (*m*_*try*_ = 2; sensitivity = 95.24; specificity = 0.00; Fig 2). However, after including SNPs in the model, accuracy increased to 0.8177 (*m*_*try*_ = 16; sensitivity = 95.24; specificity = 21.05; Fig 2). Interestingly, the model that evaluated the contribution of SNPs alone reached an accuracy of 0.8008 (m_*try*_ *=* 88; sensitivity = 36.84; specificity = 88.37; Fig 2). We show in the variance important plot (Fig 3) the 15 most important genotypes from eight genes, which presented a MDA>10 (a complete list of all genotype results is showed in S5 Table). As illustrated in Fig 3, SNP rs2472306 in *CYP1A2* was the most important variable in the prediction model (MDA = 25.3), followed by the presence of HS on MRI (MDA = 25.2).

### *ABCC2* mRNA expression in hippocampal tissue

**Fig 4:**
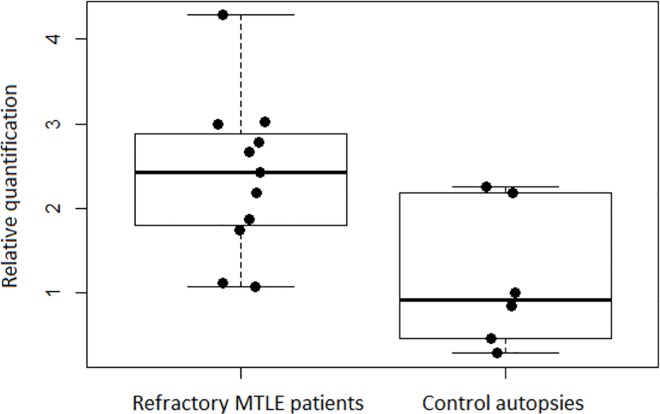
*ABCC2* mRNA relative quantification. The boxplots include dots for each sample, showing the relative quantification of ABCC2 mRNA, in terms of 2^-ΔΔCT^ (y-axis), between hippocampal tissue from refractory MTLE patients and control autopsies.

Among the top SNPs present in the best prediction model, the fourth most important variable for the model (rs2756104) is located in *ABCC2*, prompting us to analyse mRNA expression in the hippocampal tissue of patients with refractory MTLE who underwent epilepsy surgery. Our results clearly show the increased expression of *ABCC2* mRNA in hippocampal tissue from patients when compared to autopsy controls (Wilcoxon rank sum test p-value = 0.02, Fig 4).

## Discussion

We show in this study that SNP information from genes influencing drug metabolism and drug transporters, together with clinical data, can significantly improve the prediction of response to AEDs in patients with MTLE, with an estimated accuracy of 0.8177. In addition, the most important variables in the prediction model include SNPs in *ABCC2*, *CYP1A2*, *CYP1B1*, *CYP2E1*, *CYP3A4*, and *CYP3A5*, as well as *CYP2C9* and *CYP2C19*, both of which have been previously proposed as pharmacogenetic biomarkers [19].

Approximately 30.0% of patients with MTLE do not respond well to treatment with AEDs [1]. These individuals become candidates for surgical treatment after an extensive clinical investigation, which includes several trials of different combinations of AEDs. Therefore, if one could better predict which patients are less likely to respond to AED treatment, pre-operative clinical investigation could be shortened, offering a timely opportunity for patients to become seizure-free after appropriate surgical treatment. In this context, the only characteristic available to date, to be used as predictor of refractory seizures in patients with MTLE, is the presence of HS on MRI [1, 37].

Over the past few years, there has been a significant increase in the number of US-FDA approved drugs containing molecular biomarker information [19], which includes population- and genotype-specific recommended dosage. It has been proposed that genetic factors could be involved in determining the lack of response to AEDs in patients with MTLE [1, 3]. Indeed, a number of studies have shown genetic association of several SNPs in candidate genes and AED response in patients with epilepsy [14–18]. However, all of these studies were either not replicated or lacked analysis of their clinical validity, especially in comparison with the previously established predictive value of HS in discriminating refractory MTLE patients. In addition, no previous study examined simultaneously the role of SNPs in more than one candidate gene influencing AED response.

Population stratification is a real concern in population-based association studies or comparison of genotype distribution among groups. It could lead to spurious results due to allele frequency differences between groups attributable to genetic diversity in the background population, regardless of outcome status [38]. However, self-reported race in Brazilian individuals are usually associated with skin colour, and several studies have showed that skin colour is not associated with genetic structure in Brazilian population [39]. In this case, random molecular markers across the genome could be useful to evaluate differences in genetic structure between cases and controls. Furthermore, studies showed that inter-chromosomal population structure is an important confounding factor [29]. In our study, we applied additional 98 randomly chosen SNPs based on the chromosomal location of the candidate genes studied (chromosomes 2, 7, 10, 15, and 22). In addition, we performed the analysis with all five chromosomes combined and each chromosome separately. Our results showed that the between-groups variance component for all five chromosomes combined was 0.3%, while chromosome 22 separately reached 2.5%, which could be due to small number of SNPs used for the analysis. Population studies have shown that the among-groups variance component worldwide was 5.4%, ranging from 0.7% (Europe) to 3.1% (Africa) [34]. Furthermore, in this study, Fst values were lower than 0.05 for all chromosomes combined. Therefore, the two groups of patients analysed in the present study derive from the same population, allowing for unbiased comparisons between them.

There is a fundamental limitation when interpreting the results from most genetic association studies since they use hypothesis-testing approaches, which presents models that have the best descriptive features for the dataset studied, but lack predictive power to be applied to other samples [40]. Therefore, the lack of replicability in these association studies is expected, since a positive association in a sample cannot be used to predict results in another trial with a different dataset [40]. This limitation has been especially true for studies related to AED response in epilepsy. For instance, SNP rs1045642 (c.3435T>C), within *ABCB1*, was initially associated with refractory epilepsy, but several other studies could not replicate the results [16, 17]. Indeed, our study showed that SNP rs1045642 presented low importance (MDA = 5.2), to the prediction model proposed (S5 Table).

In order to overcome these limitations, we used a cross-validation approach, which optimises the parameters in a statistical model according to the analyst interest, extending the applicability of the fitted model to additional situations. In this context, we used the method to determine the parameter that maximises the discriminatory power of random forests when assessing the AED drug response in patients with MTLE. Given the strategy of splitting the dataset into two parts (training and test sets), we can apply our model to individuals that are not part of our original dataset and obtain responses that are more accurate than those provided by descriptive models. The random forests algorithm is an ensemble learning approach based on building several decision trees combined with a voting system to perform classifications. It is an improvement of decision trees as it overcomes the problem of overfitting [35]. Therefore, the LOOCV approach with random forests algorithm enabled us to identify the model with the best discriminatory properties to identify patients with AED refractory MTLE. Applying these to our dataset showed that the best parameters for discriminating patients with poor response to AEDs include the presence of HS detected by MRI and genotypic information from 11 candidate genes. In addition, we demonstrated that factors such as gender, antecedent of febrile seizures, and age of seizure onset are not present in the best prediction model. Although we did not replicate these results in a second cohort, the LOOCV statistical approach is able to minimize this limitation. Indeed, one recent review showed that, among 55 prediction studies, nine used cross-validation approach as independent validation, and only six validated in a second cohort [41]. In addition, it would be interesting to know whether specific SNPs may be associated with AED response for different drugs. However, our study was underpowered to determine whether such specific correlation exists.

Several studies have observed an improvement of discriminatory accuracy when combining clinical variables with SNP information [41], including breast cancer(from 0.58 to 0.61) [42], nasopharyngeal carcinoma (from 0.68 to 0.74) [43], and venous thrombosis (from 0.77 to 0.82)[44]. In our study, we demonstrate that by including SNP information in addition to only clinical and imaging parameters, there was a significant increase in the discriminatory accuracy from 0.4568 to 0.8177. This notable increase of the discriminatory accuracy could be due to our focus on the specific seizure aetiology (MTLE), which is an advantage under a study design where different epilepsy phenotypes are mixed. In this case, we could hypothesize that the mechanisms controlling drug response could be different in different forms of epilepsies, and this line of study could provide new insights into mechanisms of drug resistance. Although additional clinical covariates could be incorporated in prediction models, those used in our study are the ones usually most easily available to clinicians.

Genotyping kits for specific alleles in *CYP2C19* and *CYP2D6* genes have been approved by the US-FDA to be used as pharmacogenetic biomarkers (www.fda.gov) [19]. However, SNPs in *CYP2C9* and *CYP2D6* [19] present an average MAF = 0.06, significantly limiting their use in clinical practice, since they can only be detected in a small fraction of the population. In contrast, the SNP set found in our model has an average MAF = 0.20, setting a clear advantage for its use in clinical practice, mainly because this average MAF indicates that our SNP set presents a high heterozygosity, which could be used in small sample sizes.

Previous studies have described an increase in multiple drug expression genes in patients with refractory epilepsy and MTLE patients [6, 7]. We also observed an increase in *ABCC2* gene expression in hippocampal tissue from MTLE patients, compared with control autopsies. Therefore, our study strengthens the hypothesis that *ABCC2* could influence AED drug response in patients with MTLE.

In conclusion, we have shown that clinical variables in combination with SNP genotypes located in 11 genes influencing drug transport and metabolism can more accurately predict the response to drug therapy in patients with MTLE. Applying this model in clinical practice would allow a patient likely to develop resistance to treatment with AEDs to be identified sooner, thus benefiting from an earlier indication of epilepsy surgery. To the best of our knowledge, we demonstrated for the first time that genetic information improves the prediction of response to AED treatment in patients with MTLE, and could be used in clinical practice for decision-making.

## Supporting Information

S1 Table AEDs used in the past and currently for each patient on monotherapy (n = 59). Current treatment information was acquired based on pre-operative data for refractory patients who underwent surgery.
(DOC)

S2 Table AEDs used in the past and currently for each patient on polytherapy (n = 178). Current treatment information was acquired based on pre-operative data for refractory patients who underwent surgery.
(DOC)

S3 Table Allele frequency and Hardy-Weinberg disequilibrium information. We show the 119 selected SNPs for prediction analysis.
(DOC)

S4 Table Allele frequency and Hardy-Weinberg disequilibrium information. We show here the 98 selected SNPs for population stratification.
(DOC)

S5 Table Important variable table. This table shows the variables with their respective gene location and mean decrease accuracy values.
(DOC)
