# Supplementary material for: A Prediction Algorithm for Drug Response in Patients with Mesial Temporal Lobe Epilepsy Based on Clinical and Genetic Information
Source: PLoS One. 2017 Jan 4;12(1):e0169214. doi: 10.1371/journal.pone.0169214 (PMC5215688; doi:10.1371/journal.pone.0169214)
Supplement: S1 Table — Current treatment information was acquired based on pre-operative data for refractory patients who underwent surgery. (DOC) [file pone.0169214.s001.doc]

**S1 Table. AEDs used in the past and currently for each patient on monotherapy (n=59).** Current treatment information was acquired based on pre-operative data for refractory patients who underwent surgery.

| **Sample ID** | **Phenotype** | **Past treatment** | | | | | | | | | **Current treatment** |
| --- | --- | --- | --- | --- | --- | --- | --- | --- | --- | --- | --- |
|  |  | **PB** | **CBZ** | **CLB** | **PHT** | **LMT** | **VPA** | **OXC** | **TPM** | **CNZ** |  |
| 1 | refractory | - | yes | - | - | - | - | - | - | - | CBZ |
| 2 | responsive | yes | - | - | - | - | - | - | - | - | PB |
| 3 | responsive | - | yes | - | - | - | - | - | - | - | CBZ |
| 4 | responsive | - | yes | - | - | - | - | - | - | - | CBZ |
| 5 | responsive | - | yes | - | - | - | - | - | - | - | CBZ |
| 6 | responsive | - | yes | - | - | - | - | - | - | - | CBZ |
| 7 | refractory | - | yes | - | - | - | - | - | - | - | CBZ |
| 8 | refractory | - | yes | - | - | - | - | - | - | - | CBZ |
| 9 | responsive | - | yes | - | - | - | - | - | - | - | CBZ |
| 10 | refractory | - | yes | - | - | - | - | - | - | - | CBZ |
| 11 | responsive | yes | - | - | - | - | - | - | - | - | PB |
| 12 | responsive | - | yes | - | - | - | - | - | - | - | CBZ |
| 13 | refractory | - | yes | - | - | - | - | - | - | - | CBZ |
| 14 | refractory | - | yes | - | - | - | - | - | - | - | CBZ |
| 15 | refractory | - | yes | - | - | - | - | - | - | - | CBZ |
| 16 | responsive | - | yes | - | - | - | - | - | - | - | CBZ |
| 17 | responsive | - | yes | - | - | - | - | - | - | - | CBZ |
| 18 | responsive | - | yes | - | - | - | - | - | - | - | CBZ |
| 19 | responsive | - | yes | - | - | - | - | - | - | - | CBZ |
| 20 | responsive | - | - | - | yes | - | - | - | - | - | PHT |
| 21 | responsive | - | yes | - | - | - | - | - | - | - | CBZ |
| 22 | responsive | - | yes | - | - | - | - | - | - | - | CBZ |
| 23 | refractory | - | yes | - | - | - | - | - | - | - | CBZ |
| 24 | responsive | - | - | - | yes | - | - | - | - | - | PHT |
| 25 | refractory | - | yes | - | - | - | - | - | - | - | CBZ |
| 26 | responsive | - | yes | - | - | - | - | - | - | - | CBZ |
| 27 | refractory | - | yes | - | - | - | - | - | - | - | CBZ |
| 28 | refractory | - | yes | - | - | - | - | - | - | - | CBZ |
| 29 | refractory | - | yes | - | - | - | - | - | - | - | CBZ |
| 30 | responsive | yes | - | - | - | - | - | - | - | - | PB |
| 31 | responsive | - | yes | - | - | - | - | - | - | - | CBZ |
| 32 | responsive | - | yes | - | - | - | - | - | - | - | CBZ |
| 33 | refractory | - | yes | - | - | - | - | - | - | - | CBZ |
| 34 | refractory | - | yes | - | - | - | - | - | - | - | CBZ |
| 35 | refractory | - | yes | - | - | - | - | - | - | - | CBZ |
| 36 | refractory | - | yes | - | - | - | - | - | - | - | CBZ |
| 37 | refractory | - | yes | - | - | - | - | - | - | - | CBZ |
| 38 | refractory | - | yes | - | - | - | - | - | - | - | CBZ |
| 39 | responsive | - | yes | - | - | - | - | - | - | - | CBZ |
| 40 | refractory | - | yes | - | - | - | - | - | - | - | CBZ |
| 41 | refractory | - | yes | - | - | - | - | - | - | - | CBZ |
| 42 | responsive | - | yes | - | - | - | - | - | - | - | CBZ |
| 43 | responsive | yes | - | - | - | - | - | - | - | - | CBZ |
| 44 | responsive | yes | - | - | - | - | - | - | - | - | CBZ |
| 45 | responsive | - | - | - | yes | - | - | - | - | - | PHT |
| 46 | responsive | - | yes | - | - | - | - | - | - | - | CBZ |
| 47 | responsive | - | yes | - | - | - | - | - | - | - | CBZ |
| 48 | refractory | - | yes | - | - | - | - | - | - | - | CBZ |
| 49 | refractory | - | yes | - | - | - | - | - | - | - | CBZ |
| 50 | refractory | - | yes | - | - | - | - | - | - | - | CBZ |
| 51 | refractory | - | yes | - | - | - | - | - | - | - | CBZ |
| 52 | refractory | - | yes | - | - | - | - | - | - | - | CBZ |
| 53 | refractory | - | yes | - | - | - | - | - | - | - | CBZ |
| 54 | refractory | - | yes | - | - | - | - | - | - | - | CBZ |
| 55 | responsive | - | yes | - | - | - | - | - | - | - | CBZ |
| 56 | refractory | - | yes | - | - | - | - | - | - | - | CBZ |
| 57 | refractory | - | yes | - | - | - | - | - | - | - | CBZ |
| 58 | responsive | - | - | - | yes | - | - | - | - | - | PHT |
| 59 | responsive | - | yes | - | - | - | - | - | - | - | CBZ |

PB, phenobarbital; CBZ, carbamazepine; CLB, clobazan; PHT, phenytoin; LMT, lamotrigine; VPA, valproic acid; OXC, oxcarbazepine; TPM, topiramate; CNZ, clonazepam; DZP, diazepam.
