# Supplementary material for: A Prediction Algorithm for Drug Response in Patients with Mesial Temporal Lobe Epilepsy Based on Clinical and Genetic Information
Source: PLoS One. 2017 Jan 4;12(1):e0169214. doi: 10.1371/journal.pone.0169214 (PMC5215688; doi:10.1371/journal.pone.0169214)
Supplement: S2 Table — Current treatment information was acquired based on pre-operative data for refractory patients who underwent surgery. (DOC) [file pone.0169214.s002.doc]

**S2 Table. AEDs used in the past and currently for each patient on polytherapy (n=178).** Current treatment information was acquired based on pre-operative data for refractory patients who underwent surgery.

| **Sample ID** | **Phenotype** | **Past treatment** | | | | | | | | | **Current treatment** |
| --- | --- | --- | --- | --- | --- | --- | --- | --- | --- | --- | --- |
|  |  | **PB** | **CBZ** | **CLB** | **PHT** | **LMT** | **VPA** | **OXC** | **TPM** | **CNZ** |  |
| 60 | responsive | yes | yes | - | yes | - | yes | - | - | - | CBZ |
| 61 | responsive | yes | yes | - | yes | - | - | - | - | - | PB, CBZ, PHT |
| 62 | responsive | - | - | - | yes | yes | - | - | - | - | PHT, LMT |
| 63 | refractory | yes | yes | - | yes | - | - | - | - | - | CBZ, CLB |
| 64 | responsive | yes | yes | - | yes | - | - | - | - | - | CLB, LMT, VPA |
| 65 | refractory | - | - | yes | yes | - | - | - | - | - | PHT, CNZ |
| 66 | responsive | yes | yes | - | yes | - | - | - | - | - | withoud AEDs |
| 67 | refractory | yes | yes | - | - | - | - | - | - | - | PB, CBZ |
| 68 | refractory | yes | yes | yes | - | - | yes | yes | - | - | CBZ, PHT, CLB |
| 69 | responsive | - | yes | yes | - | - | - | - | - | - | CBZ, CLB |
| 70 | refractory | yes | yes | yes | - | - | - | - | - | - | CBZ, CLB |
| 71 | responsive | - | yes | yes | - | - | - | - | - | - | CBZ, CLB |
| 72 | refractory | yes | yes | - | yes | - | yes | - | - | - | CBZ, CLB |
| 73 | refractory | yes | yes | yes | yes | yes | yes | - | - | - | CLB, LMT, VPA |
| 74 | responsive | - | yes | yes | - | - | yes | - | - | - | CBZ, CLB |
| 75 | refractory | - | yes | - | yes | - | - | - | - | - | CBZ, CLB |
| 76 | refractory | yes | - | - | yes | - | - | - | - | - | CBZ |
| 77 | refractory | yes | yes | - | yes | - | - | - | - | - | CBZ.CLB, VPA |
| 78 | responsive | - | yes | yes | yes | - | yes | - | - | - | CBZ, CLB |
| 79 | refractory | - | yes | yes | - | - | - | - | - | - | CBZ, CLB |
| 80 | refractory | - | yes | yes | - | - | - | - | - | - | CBZ, CLB |
| 81 | refractory | - | yes | yes | yes | - | - | - | - | - | CBZ, CLB |
| 82 | responsive | - | - | yes | - | yes | yes | - | - | - | CLB, LMT, VPA |
| 83 | refractory | - | yes | yes | - | - | - | - | - | - | CBZ, CLB |
| 84 | refractory | - | yes | yes | yes | - | - | - | - | - | CBZ, CLB |
| 85 | refractory | - | yes | yes | - | - | yes | - | - | - | CBZ, CLB, VPA |
| 86 | refractory | - | yes | yes | - | - | - | - | - | - | CBZ, CLB |
| 87 | refractory | - | yes | yes | - | - | - | yes | - | - | CBZ, CLB |
| 88 | refractory | yes | yes | - | yes | - | - | - | - | - | CBZ, CLB |
| 89 | refractory | yes | yes | - | yes | - | - | - | - | - | CBZ |
| 90 | responsive | yes | yes | - | yes | - | yes | - | - | - | CBZ |
| 91 | responsive | yes | yes | yes | - | - | - | - | - | - | CBZ, CLB |
| 92 | refractory | yes | yes | - | yes | - | - | - | - | - | CBZ, CLB |
| 93 | refractory | yes | yes | yes | yes | - | yes | - | - | - | CBZ |
| 94 | refractory | - | yes | yes | - | - | yes | - | - | - | CBZ, CLB, VPA |
| 95 | refractory | yes | - | - | yes | - | - | - | - | - | PB, PHT |
| 96 | refractory | yes | yes | - | yes | - | - | - | - | - | CBZ |
| 97 | refractory | yes | yes | - | yes | - | - | - | - | - | CBZ |
| 98 | refractory | - | yes | - | yes | yes | - | - | - | - | CBZ |
| 99 | refractory | yes | yes | - | - | - | yes | - | - | - | VPA |
| 100 | refractory | yes | yes | - | yes | - | - | - | - | - | CBZ, CLB |
| 101 | refractory | yes | yes | - | yes | - | - | - | - | - | CBZ, CLB, LMT |
| 102 | refractory | yes | yes | - | yes | - | - | - | - | - | CBZ, DZP |
| 103 | refractory | yes | yes | yes | yes | - | - | - | - | - | CBZ, CLB |
| 104 | refractory | yes | yes | - | yes | - | - | - | - | - | CBZ, CLB |
| 105 | responsive | yes | yes | - | yes | - | - | - | - | - | PB, CBZ, PHT |
| 106 | refractory | yes | yes | yes | yes | - | yes | - | - | - | CBZ, CLB |
| 107 | refractory | - | yes | yes | - | - | - | - | - | - | CBZ, CLB |
| 108 | refractory | - | yes | - | yes | - | - | - | - | - | CBZ, CLB |
| 109 | refractory | yes | yes | yes | yes | - | yes | - | - | - | CBZ, CLB |
| 110 | refractory | yes | yes | - | yes | - | - | - | - | - | CBZ, CLB |
| 111 | responsive | yes | yes | - | yes | - | - | - | - | - | CBZ |
| 112 | refractory | - | yes | yes | yes | - | yes | - | yes | - | CBZ |
| 113 | refractory | - | yes | yes | - | - | - | - | yes | - | CBZ, CLB |
| 114 | refractory | - | yes | yes | - | - | - | - | - | - | CBZ, CLB |
| 115 | refractory | yes | yes | yes | yes | - | - | - | - | - | PHT, CLB |
| 116 | refractory | yes | yes | yes | - | yes | yes | - | - | - | CBZ, CLB, LMT |
| 117 | refractory | - | - | yes | - | - | - | yes | - | - | CLB, OXC |
| 118 | refractory | yes | yes | yes | - | - | - | - | - | - | CBZ, CLB |
| 119 | refractory | - | yes | - | yes | - | - | - | - | - | CBZ, CLB |
| 120 | refractory | - | yes | yes | - | - | - | - | - | - | CBZ, CLB |
| 121 | refractory | yes | yes | - | yes | - | - | - | - | - | CBZ |
| 122 | refractory | yes | yes | yes | - | - | yes | - | - | - | CBZ, CLB |
| 123 | responsive | yes | yes | - | yes | - | - | - | - | - | CBZ |
| 124 | responsive | yes | yes | - | yes | - | - | - | - | - | PHT |
| 125 | refractory | yes | yes | - | yes | - | - | - | - | - | CBZ, CLB, LMT |
| 126 | refractory | - | yes | yes | - | - | - | - | - | - | CBZ, CLB |
| 127 | refractory | yes | yes | - | yes | - | - | - | - | - | CBZ |
| 128 | responsive | - | yes | yes | - | - | - | - | yes | - | CBZ, CLB, TPM |
| 129 | refractory | yes | yes | - | yes | - | - | - | - | - | CBZ, CLB |
| 130 | refractory | - | yes | yes | - | - | - | - | yes | - | CBZ, CLB, TPM |
| 131 | refractory | yes | yes | - | yes | - | - | - | - | - | CBZ |
| 132 | refractory | yes | yes | - | yes | - | - | - | - | - | CBZ, CLB, TPM |
| 133 | refractory | yes | yes | - | yes | - | - | - | - | - | PHT |
| 134 | responsive | yes | yes | - | yes | - | - | - | - | - | PB, CBZ, PHT |
| 135 | refractory | - | yes | yes | - | - | - | - | - | - | CBZ, CLB |
| 136 | refractory | yes | yes | - | - | - | yes | - | - | - | CBZ, CLB |
| 137 | refractory | - | yes | yes | - | yes | - | - | - | - | CBZ, CLB, LMT |
| 138 | refractory | - | yes | yes | - | - | - | - | - | - | CBZ, CLB |
| 139 | refractory | - | yes | yes | - | - | - | - | - | - | CBZ, CLB |
| 140 | refractory | yes | yes | - | - | - | - | - | - | - | PHT, CLB |
| 141 | refractory | yes | yes | - | yes | - | - | - | - | - | CBZ, CLB, TPM |
| 142 | refractory | yes | yes | - | yes | - | - | - | - | - | CLB, LMT, OXC |
| 143 | refractory | - | yes | yes | - | - | - | - | - | - | CBZ., CLB |
| 144 | responsive | yes | yes | - | yes | - | - | - | - | - | CLB, PHT |
| 145 | responsive | - | - | yes | yes | - | - | - | - | - | CLB, PHT |
| 146 | responsive | - | yes | - | - | - | - | - | yes | - | TPM |
| 147 | refractory | yes | yes | - | yes | - | - | - | - | - | CBZ |
| 148 | responsive | yes | yes | yes | yes | - | - | - | - | - | CLB, PHT |
| 149 | refractory | yes | - | yes | yes | - | yes | yes | yes | yes | TPM |
| 150 | refractory | - | yes | - | - | yes | - | - | - | - | CBZ, LMT |
| 151 | responsive | yes | - | - | yes | - | - | - | - | - | PB, PHT |
| 152 | refractory | - | - | yes | - | - | - | yes | - | - | CLB, OXC |
| 153 | responsive | - | yes | yes | - | - | yes | - | - | - | CBZ, CLB |
| 154 | refractory | yes | yes | - | - | - | yes | - | - | - | PB |
| 155 | refractory | yes | yes | - | yes | - | - | - | - | - | CBZ, CLB, LMT |
| 156 | refractory | yes | yes | yes | - | - | - | - | - | - | CBZ, CLB |
| 157 | refractory | yes | yes | - | - | yes | - | - | - | - | CBZ |
| 158 | refractory | yes | yes | yes | - | - | - | - | - | - | PHT |
| 159 | refractory | yes | yes | - | - | - | - | - | - | - | CBZ |
| 160 | refractory | yes | yes | - | - | - | - | - | - | - | CBZ |
| 161 | refractory | - | yes | yes | - | - | - | - | - | - | CBZ, LMT |
| 162 | refractory | - | yes | - | yes | - | - | - | - | - | CBZ, CLB |
| 163 | refractory | - | yes | - | - | - | yes | - | - | - | CBZ, VPA |
| 164 | refractory | yes | - | yes | - | - | - | - | - | - | CBZ, CLB |
| 165 | responsive | yes | yes | - | yes | - | - | - | - | - | CBZ |
| 166 | refractory | - | yes | yes | - | - | - | - | - | - | CBZ, CLB |
| 167 | refractory | yes | yes | - | yes | - | - | - | - | - | PB, CBZ, PHT |
| 168 | responsive | yes | - | - | yes | - | - | - | - | - | PB, PHT |
| 169 | refractory | - | yes | yes | - | - | - | - | yes | - | CBZ, CLB |
| 170 | responsive | - | yes | yes | - | - | - | - | - | - | CBZ, CLB |
| 171 | refractory | yes | - | yes | - | - | - | - | - | - | CBZ |
| 172 | refractory | - | yes | yes | - | - | yes | - | - | - | CBZ |
| 173 | refractory | yes | yes | yes | yes | - | - | - | - | - | CLB, PHT |
| 174 | responsive | - | yes | - | yes | - | yes | - | - | - | CBZ |
| 175 | refractory | - | yes | yes | - | - | - | - | - | - | CBZ, CLB |
| 176 | refractory | - | yes | yes | - | - | - | - | - | - | CLB, TPM |
| 177 | refractory | yes | yes | - | yes | - | - | - | - | - | CBZ, TPM |
| 178 | responsive | - | yes | - | yes | - | - | - | - | - | CBZ, PHT |
| 179 | refractory | yes | yes | - | - | - | - | - | - | - | CBZ, CLB |
| 180 | refractory | - | yes | yes | - | - | - | - | - | - | CBZ, CLB |
| 181 | refractory | - | yes | - | yes | - | - | - | - | - | CBZ, CLB |
| 182 | responsive | - | yes | yes | yes | - | yes | - | - | - | CBZ |
| 183 | responsive | - | yes | - | - | - | - | - | yes | - | CBZ, CLB, TPM |
| 184 | refractory | yes | yes | - | yes | - | yes | - | - | - | TPM, CNZ |
| 185 | responsive | - | yes | yes | - | - | - | - | - | - | CBZ, CLB |
| 186 | responsive | - | yes | yes | - | - | - | - | - | - | CBZ, CLB |
| 187 | refractory | yes | yes | - | yes | - | - | - | - | - | CBZ, CLB |
| 188 | refractory | yes | yes | - | yes | - | - | - | yes | - | CBZ, TPM |
| 189 | refractory | - | yes | yes | - | - | - | - | - | - | CBZ, CLB |
| 190 | refractory | - | yes | yes | - | - | - | - | - | - | CBZ, CLB |
| 191 | refractory | yes | yes | - | yes | - | - | - | - | - | CBZ, CLB |
| 192 | refractory | yes | yes | - | yes | - | - | - | - | - | CBZ, CLB, CNZ |
| 193 | responsive | yes | yes | - | - | - | - | - | - | yes | PB, CBZ, CNZ |
| 194 | responsive | yes | - | - | yes | - | - | - | - | - | PB |
| 195 | responsive | yes | - | - | - | - | yes | - | - | - | PB, PHT |
| 196 | refractory | yes | yes | - | yes | - | - | - | - | - | PB, PHT |
| 197 | refractory | yes | yes | - | yes | - | - | - | - | - | CBZ |
| 198 | refractory | yes | yes | yes | yes | - | yes | - | - | - | PHT, OXC, CNZ |
| 199 | refractory | yes | - | yes | yes | yes | yes | - | - | - | CLB, LMT, VPA |
| 200 | refractory | - | yes | yes | - | - | - | - | - | - | CBZ |
| 201 | refractory | - | - | yes | - | yes | yes | - | - | - | CLB, LMT, VPA |
| 202 | responsive | - | yes | - | yes | - | - | - | - | - | CBZ |
| 203 | refractory | yes | - | - | yes | - | - | - | - | - | CBZ, CLB, VPA |
| 204 | refractory | yes | - | - | yes | - | - | - | - | - | VPA |
| 205 | refractory | yes | yes | yes | - | - | - | - | yes | - | CBZ, NTZ |
| 206 | refractory | yes | yes | - | yes | - | - | - | - | - | CBZ |
| 207 | refractory | - | yes | yes | - | - | - | - | - | - | CBZ, CLB, LMT |
| 208 | refractory | - | yes | - | yes | yes | - | - | - | - | LMT |
| 209 | responsive | yes | yes | yes | - | - | - | - | - | - | CBZ |
| 210 | responsive | yes | yes | - | yes | - | - | - | - | yes | PB, CLB, DZP |
| 211 | refractory | - | yes | yes | - | - | - | - | yes | - | CBZ, CLB, TPM |
| 212 | refractory | yes | yes | yes | yes | - | - | - | - | - | CBZ, CLB |
| 213 | refractory | yes | yes | - | yes | - | - | - | - | - | CBZ, CLB |
| 214 | refractory | yes | yes | - | yes | - | - | - | - | - | LMT, VPA |
| 215 | refractory | - | yes | yes | yes | - | yes | yes | yes | - | CBZ, CLB |
| 216 | refractory | yes | yes | - | yes | - | - | - | - | - | CBZ |
| 217 | refractory | yes | yes | yes | yes | - | - | - | - | - | CLB, CBZ |
| 218 | refractory | - | yes | yes | - | - | - | - | yes | - | CBZ, CLB, TPM |
| 219 | refractory | yes | yes | - | - | - | - | - | - | yes | CBZ |
| 220 | responsive | - | - | yes | - | yes | - | - | - | - | CLB, LMT |
| 221 | refractory | - | yes | - | yes | - | - | - | - | - | CBZ, CLB |
| 222 | responsive | yes | yes | - | - | - | - | - | - | - | PB, CBZ |
| 223 | refractory | yes | yes | - | - | - | - | - | yes | - | OXC, LEV, LAC |
| 224 | refractory | - | yes | yes | - | - | - | - | - | - | CBZ, CLB |
| 225 | refractory | - | yes | yes | - | - | - | - | yes | - | CBZ |
| 226 | responsive | yes | - | - | yes | - | - | - | - | - | withoud AEDs |
| 227 | responsive | - | - | yes | yes | - | - | - | - | - | CLB, PHT |
| 228 | refractory | yes | yes | yes | yes | - | yes | - | - | - | CBZ, CLB |
| 229 | refractory | - | yes | - | yes | - | - | - | - | - | CBZ, CLB |
| 230 | refractory | - | yes | yes | - | - | - | - | - | - | CBZ, CLB |
| 231 | refractory | - | yes | yes | - | - | - | - | - | - | CBZ, CLB |
| 232 | responsive | - | yes | - | yes | - | - | - | - | - | CBZ, CLB |
| 233 | responsive | - | yes | yes | yes | - | - | - | - | - | CBZ |
| 234 | refractory | - | yes | yes | - | yes | - | - | - | - | CLB, LMT |
| 235 | refractory | - | yes | - | yes | - | - | - | - | - | CBZ |
| 236 | refractory | yes | yes | yes | yes | - | - | yes | - | - | CBZ, CLB |
| 237 | refractory | yes | yes | yes | - | - | - | - | - | - | PB, CBZ, PHT |

PB, phenobarbital; CBZ, carbamazepine; CLB, clobazan; PHT, phenytoin; LMT, lamotrigine; VPA, valproic acid; OXC, oxcarbazepine; TPM, topiramate; CNZ, clonazepam, DZP, diazepam; LEV, levetiracetam; NTZ, nitrazepam; LAC, lacosamide.
