# Supplementary material for: A Prediction Algorithm for Drug Response in Patients with Mesial Temporal Lobe Epilepsy Based on Clinical and Genetic Information
Source: PLoS One. 2017 Jan 4;12(1):e0169214. doi: 10.1371/journal.pone.0169214 (PMC5215688; doi:10.1371/journal.pone.0169214)
Supplement: S3 Table — We show the 119 selected SNPs for prediction analysis. (DOC) [file pone.0169214.s003.doc]

**S3 Table. Allele frequency and Hardy-Weinberg disequilibrium information. We show the 119 selected SNPs for prediction analysis.**

| **Name** | **Allele** | **MAF** | **HWD p-value** | **Genotype call rate (%)** |
| --- | --- | --- | --- | --- |
| rs2229109 | T | 0.049 | 0.8337 | 99.6 |
| rs1128503 | A | 0.348 | 0.6511 | 99.1 |
| rs1045642 | A | 0.377 | 0.5213 | 99.1 |
| rs2235048 | G | 0.368 | 0.6599 | 98.7 |
| rs717620 | T | 0.186 | 0.9116 | 88.1 |
| rs2756104 | T | 0.430 | 0.7902 | 97.3 |
| rs2273697 | A | 0.174 | 0.9806 | 96.9 |
| rs2002042 | T | 0.253 | 0.3783 | 96.9 |
| rs4148396 | T | 0.335 | 0.2271 | 89.8 |
| rs3758395 | C | 0.047 | 0.1322 | 94.7 |
| rs3740067 | G | 0.384 | 1.0000 | 96.9 |
| rs17216317 | T | 0.085 | 0.1074 | 96.0 |
| rs3740066 | T | 0.366 | 0.4094 | 97.3 |
| rs3740065 | G | 0.136 | 0.4080 | 96.0 |
| rs1137968 | T | 0.082 | 0.8326 | 94.7 |
| rs8187710 | A | 0.100 | 0.7116 | 94.7 |
| rs162549 | T | 0.263 | 1.0000 | 88.5 |
| rs9341263 | G | 0.209 | 0.3104 | 91.2 |
| rs162562 | C | 0.225 | 0.4630 | 91.6 |
| rs162561 | A | 0.174 | 0.6483 | 84.1 |
| rs2551188 | T | 0.356 | 0.5000 | 88.9 |
| rs9341244 | T | 0.071 | 0.6893 | 90.7 |
| rs2404955 | A | 0.218 | 0.1299 | 91.2 |
| rs6956344 | T | 0.133 | 0.4844 | 95.1 |
| rs4646437 | T | 0.244 | 0.7918 | 88.9 |
| rs28988579 | G | 0.058 | 1.0000 | 91.6 |
| rs12114000 | A | 0.042 | 0.6175 | 95.6 |
| rs776746 | A | 0.196 | 0.9286 | 90.3 |
| rs28365067 | T | 0.071 | 0.5484 | 93.8 |
| rs28371764 | T | 0.082 | 1.0000 | 94.7 |
| rs12768009 | A | 0.139 | 1.0000 | 93.8 |
| rs4986893 | A | 0.096 | 0.6008 | 94.2 |
| rs2104161 | T | 0.311 | 0.2259 | 91.2 |
| rs12253253 | C | 0.188 | 0.0316 | 95.6 |
| rs2253635 | G | 0.367 | 0.6325 | 92.9 |
| rs7089580 | T | 0.202 | 0.0531 | 87.6 |
| rs4086116 | T | 0.129 | 0.2211 | 94.7 |
| rs4918766 | A | 0.421 | 0.4227 | 92.0 |
| rs2153628 | G | 0.188 | 0.1923 | 95.1 |
| rs9332172 | G | 0.199 | 0.8618 | 92.5 |
| rs1934963 | C | 0.213 | 0.1154 | 94.7 |
| rs1057910 | C | 0.062 | 1.0000 | 92.5 |
| rs2515641 | T | 0.207 | 0.2793 | 92.9 |
| rs915906 | C | 0.249 | 0.3410 | 94.2 |
| rs8192772 | C | 0.094 | 0.5503 | 93.8 |
| rs6413419 | A | 0.066 | 0.1002 | 93.4 |
| rs915907 | A | 0.212 | 0.9306 | 92.9 |
| rs8192775 | A | 0.127 | 0.4424 | 92.0 |
| rs7092584 | T | 0.164 | 0.1478 | 93.4 |
| rs743535 | T | 0.102 | 0.6997 | 91.2 |
| rs2070677 | T | 0.256 | 0.4548 | 91.6 |
| rs2515644 | A | 0.298 | 0.1427 | 95.1 |
| rs4986882 | G | 0.076 | 0.5361 | 96.0 |
| rs1799814 | A | 0.137 | 0.3059 | 90.3 |
| rs17861094 | G | 0.132 | 0.2086 | 88.9 |
| rs4646421 | T | 0.232 | 0.9203 | 93.4 |
| rs8031941 | G | 0.069 | 0.1092 | 90.3 |
| rs2069526 | G | 0.084 | 1.0000 | 94.7 |
| rs12904742 | A | 0.075 | 0.5979 | 92.0 |
| rs28360521 | T | 0.163 | 0.9796 | 95.1 |
| rs10264272 | T | 0.028 | 0.2672 | 87.2 |
| rs1056836 | G | 0.435 | 0.0369 | 88.5 |
| rs1080985 | G | 0.275 | 0.0421 | 91.6 |
| rs11188092 | C | 0.203 | 0.1069 | 93.8 |
| rs11568732 | G | 0.100 | 0.0235 | 96.9 |
| rs11597626 | G | 0.174 | 0.0144 | 91.6 |
| rs1200313 | G | 0.332 | 0.1077 | 86.7 |
| rs12268020 | T | 0.195 | 0.0183 | 92.9 |
| rs12333983 | A | 0.153 | 7.00E-04 | 89.8 |
| rs12571421 | G | 0.162 | 0.0019 | 87.2 |
| rs12773342 | C | 0.269 | 0.2315 | 91.2 |
| rs12778026 | A | 0.170 | 0.0135 | 95.1 |
| rs1322179 | A | 0.141 | 0.7652 | 92.5 |
| rs1505 | C | 0.456 | 1.74E-08 | 94.7 |
| rs1555474 | C | 0.403 | 0.2143 | 88.5 |
| rs17222632 | C | 0.003 | 1.0000 | 85.4 |
| rs17222723 | A | 0.071 | 0.9023 | 69.0 |
| rs1800440 | G | 0.286 | 2.39E-06 | 89.8 |
| rs1856908 | T | 0.179 | 2.26E-07 | 91.6 |
| rs2032586 | G | 0.000 | 1.0000 | 86.3 |
| rs2070673 | A | 0.050 | 0.1833 | 96.5 |
| rs2073336 | A | 0.500 | 4.91E-52 | 77.9 |
| rs2235039 | G | 0.000 | 1.0000 | 85.8 |
| rs2249694 | A | 0.302 | 0.0097 | 93.8 |
| rs2256871 | C | 0.034 | 1.0000 | 96.5 |
| rs2405184 | G | 0.019 | 1.0000 | 91.2 |
| rs2470890 | T | 0.208 | 0.0029 | 93.8 |
| rs2472306 | A | 0.189 | 0.1710 | 93.8 |
| rs2475376 | T | 0.333 | 1.63E-11 | 78.3 |
| rs2515642 | C | 0.320 | 0.2559 | 91.2 |
| rs2687111 | C | 0.014 | 5.00E-04 | 93.8 |
| rs2756109 | G | 0.461 | 0.0011 | 85.0 |
| rs28365083 | A | 0.018 | 1.0000 | 96.9 |
| rs28365087 | A | 0.050 | 0.7896 | 88.1 |
| rs28371730 | A | 0.341 | 5.50E-05 | 81.9 |
| rs28399419 | T | 0.044 | 0.0091 | 95.1 |
| rs28399429 | A | 0.044 | 0.0827 | 86.3 |
| rs2855658 | A | 0.431 | 0.0027 | 93.4 |
| rs2856844 | G | 0.171 | 0.2546 | 88.1 |
| rs3213619 | C | 0.026 | 1.0000 | 86.3 |
| rs3758580 | T | 0.132 | 1.0000 | 96.9 |
| rs3758581 | A | 0.213 | 2.00E-04 | 95.6 |
| rs4304697 | A | 0.016 | 1.0000 | 95.6 |
| rs4417205 | G | 0.132 | 0.0172 | 87.2 |
| rs4617515 | A | 0.429 | 0.0324 | 96.9 |
| rs4646457 | G | 0.096 | 1.05E-05 | 89.8 |
| rs4917623 | C | 0.333 | 1.00E-04 | 95.6 |
| rs4917639 | C | 0.307 | 0.0011 | 88.1 |
| rs4918797 | T | 0.198 | 0.1111 | 89.4 |
| rs4986879 | G | 0.043 | 0.5973 | 88.5 |
| rs743534 | G | 0.250 | 0.1379 | 83.2 |
| rs762551 | C | 0.324 | 0.0080 | 96.9 |
| rs7897079 | G | 0.161 | 0.0310 | 89.4 |
| rs7916649 | G | 0.479 | 0.0859 | 85.8 |
| rs9282564 | G | 0.043 | 0.5745 | 81.9 |
| rs9332104 | C | 0.181 | 0.0392 | 94.2 |
| rs9332168 | T | 0.171 | 0.2213 | 92.0 |
| rs9332174 | G | 0.208 | 0.0338 | 87.2 |
| rs9341249 | C | 0.133 | 8.00E-04 | 88.5 |

HWD, Hardy-Weinberg disequilibrium; MAF, minimum allele frequency.
