# Supplementary material for: A Prediction Algorithm for Drug Response in Patients with Mesial Temporal Lobe Epilepsy Based on Clinical and Genetic Information
Source: PLoS One. 2017 Jan 4;12(1):e0169214. doi: 10.1371/journal.pone.0169214 (PMC5215688; doi:10.1371/journal.pone.0169214)
Supplement: S4 Table — We show here the 98 selected SNPs for population stratification. (DOC) [file pone.0169214.s004.doc]

**S4 Table. Allele frequency and Hardy-Weinberg disequilibrium information.** We show here the 98 selected SNPs for population stratification.

| **Name** | **Allele** | **MAF** | **HWD p-value** | **Genotype call rate (%)** |
| --- | --- | --- | --- | --- |
| rs10179086 | C | 0.277 | 0.8672 | 75.5 |
| rs10764125 | C | 0.203 | 0.1096 | 82.7 |
| rs10796322 | C | 0.500 | 1.66E-05 | 67.8 |
| rs10826926 | G | 0.126 | 0.4912 | 80.3 |
| rs10953607 | A | 0.358 | 0.0106 | 83.2 |
| rs11001309 | T | 0.096 | 0.7633 | 70.2 |
| rs11761505 | C | 0.303 | 0.5351 | 63.5 |
| rs11972012 | C | 0.318 | 0.7680 | 63.5 |
| rs12264577 | C | 0.020 | 1.0000 | 82.7 |
| rs13245505 | C | 0.229 | 1.0000 | 70.2 |
| rs1390720 | C | 0.263 | 0.0182 | 75.0 |
| rs1829611 | A | 0.134 | 0.0710 | 78.8 |
| rs1900505 | G | 0.280 | 4.37E-06 | 61.1 |
| rs2247981 | T | 0.282 | 0.4383 | 70.7 |
| rs2420673 | G | 0.234 | 0.0367 | 69.7 |
| rs2493624 | G | 0.222 | 0.8431 | 80.3 |
| rs2909450 | A | 0.184 | 0.6383 | 67.8 |
| rs312966 | C | 0.493 | 0.0602 | 69.2 |
| rs379768 | A | 0.362 | 0.2471 | 67.8 |
| rs4670075 | T | 0.154 | 0.4017 | 81.2 |
| rs4854183 | A | 0.193 | 0.1061 | 71.2 |
| rs6432838 | C | 0.226 | 2.56E-05 | 81.7 |
| rs6713136 | A | 0.292 | 0.0813 | 69.2 |
| rs6727767 | T | 0.280 | 0.9521 | 64.4 |
| rs6749903 | A | 0.278 | 1.05E-13 | 73.6 |
| rs6956840 | T | 0.358 | 0.4380 | 79.8 |
| rs6962710 | C | 0.371 | 0.0171 | 72.6 |
| rs7076249 | G | 0.194 | 0.0251 | 58.2 |
| rs7088472 | A | 0.268 | 8.74E-07 | 66.3 |
| rs7576924 | C | 0.468 | 1.35E-09 | 75.0 |
| rs7808534 | T | 0.299 | 0.3671 | 66.8 |
| rs7904646 | T | 0.077 | 1.0000 | 75.0 |
| rs7914287 | C | 0.261 | 0.6662 | 68.3 |
| rs8023613 | T | 0.134 | 0.0375 | 84.1 |
| rs805803 | A | 0.173 | 1.0000 | 72.1 |
| rs840706 | C | 0.218 | 1.0000 | 70.7 |
| rs873724 | T | 0.480 | 0.8246 | 73.6 |
| rs9325460 | T | 0.083 | 0.6276 | 80.8 |
| rs978854 | T | 0.314 | 0.4376 | 76.4 |
| rs10209471 | C | 0.301 | 0.6715 | 95.2 |
| rs10269564 | C | 0.140 | 0.8728 | 96.2 |
| rs11631645 | G | 0.422 | 0.2024 | 89.9 |
| rs11675509 | G | 0.206 | 0.0474 | 71.2 |
| rs11980796 | G | 0.138 | 0.0073 | 94.2 |
| rs12615074 | T | 0.364 | 1.18E-05 | 89.9 |
| rs13008910 | T | 0.120 | 0.3992 | 94.2 |
| rs13033651 | T | 0.441 | 1.56E-13 | 86.1 |
| rs13057617 | G | 0.207 | 0.8077 | 90.4 |
| rs1468163 | A | 0.418 | 0.4072 | 93.3 |
| rs1597557 | A | 0.376 | 0.7639 | 77.4 |
| rs1696839 | C | 0.258 | 0.7424 | 92.3 |
| rs17125571 | C | 0.220 | 0.2323 | 96.2 |
| rs17202235 | C | 0.234 | 0.7869 | 90.4 |
| rs1767103 | C | 0.180 | 0.0470 | 82.7 |
| rs1949737 | A | 0.497 | 0.1753 | 89.9 |
| rs2070735 | A | 0.194 | 0.1358 | 92.8 |
| rs2420936 | A | 0.455 | 1.0000 | 96.6 |
| rs265518 | T | 0.389 | 1.48E-13 | 95.7 |
| rs2817656 | G | 0.287 | 0.4834 | 97.1 |
| rs284552 | T | 0.038 | 0.4529 | 88.5 |
| rs3814205 | C | 0.209 | 0.0972 | 95.7 |
| rs4019731 | C | 0.153 | 1.30E-14 | 86.5 |
| rs412079 | C | 0.386 | 0.2647 | 94.7 |
| rs453534 | T | 0.254 | 0.8307 | 90.9 |
| rs4919098 | G | 0.132 | 1.0000 | 81.7 |
| rs5748869 | G | 0.367 | 1.87E-10 | 86.5 |
| rs5753991 | A | 0.196 | 0.4476 | 90.9 |
| rs6432729 | G | 0.354 | 0.4862 | 59.1 |
| rs6707475 | T | 0.409 | 0.8871 | 58.2 |
| rs7074356 | A | 0.062 | 5.00E-04 | 97.1 |
| rs7074960 | A | 0.339 | 0.0015 | 80.8 |
| rs7094342 | C | 0.197 | 0.0201 | 81.7 |
| rs7425100 | C | 0.015 | 1.0000 | 96.2 |
| rs7609407 | G | 0.238 | 0.5946 | 92.8 |
| rs7894703 | C | 0.256 | 0.0197 | 81.7 |
| rs7903331 | T | 0.113 | 0.4805 | 97.6 |
| rs801758 | A | 0.203 | 2.89E-15 | 89.9 |
| rs867354 | C | 0.389 | 0.0116 | 84.6 |
| rs9615812 | A | 0.143 | 0.0039 | 82.2 |
| rs10235911 | C | 0.427 | 0.0267 | 98.6 |
| rs10250100 | G | 0.149 | 0.5488 | 98.6 |
| rs10274403 | G | 0.215 | 0.2601 | 96.2 |
| rs11072380 | T | 0.261 | 0.6280 | 98.6 |
| rs12901781 | A | 0.316 | 0.8045 | 98.1 |
| rs2185311 | C | 0.400 | 0.8097 | 98.6 |
| rs2195114 | A | 0.337 | 0.0154 | 98.6 |
| rs2742236 | A | 0.449 | 0.0082 | 98.6 |
| rs343085 | G | 0.224 | 8.00E-04 | 96.6 |
| rs4331020 | A | 0.437 | 0.0027 | 98.6 |
| rs4636605 | G | 0.267 | 0.7016 | 98.1 |
| rs4774602 | A | 0.473 | 0.7836 | 98.1 |
| rs6547762 | T | 0.211 | 1.07E-08 | 98.1 |
| rs6716554 | C | 0.288 | 0.6733 | 97.6 |
| rs6966622 | T | 0.256 | 0.9074 | 97.6 |
| rs6970237 | G | 0.215 | 0.0915 | 98.6 |
| rs7073142 | C | 0.180 | 0.6186 | 98.6 |
| rs7568053 | A | 0.339 | 0.9832 | 98.6 |
| rs975789 | T | 0.051 | 1.0000 | 98.6 |

HWD, Hardy-Weinberg disequilibrium; MAF, minimum allele frequency
