# Supplementary material for: A Prediction Algorithm for Drug Response in Patients with Mesial Temporal Lobe Epilepsy Based on Clinical and Genetic Information
Source: PLoS One. 2017 Jan 4;12(1):e0169214. doi: 10.1371/journal.pone.0169214 (PMC5215688; doi:10.1371/journal.pone.0169214)
Supplement: S5 Table — This table shows the variables with their respective gene location and mean decrease accuracy values. (DOC) [file pone.0169214.s005.doc]

**S5 Table. Important variable table.** This table shows the variables with their respective gene location and mean decrease accuracy values.

| **Variable** | **Gene** | **MDA** |
| --- | --- | --- |
| rs2472306AB | *CYP1A2* | 25.3 |
| Presence of HS | *-* | 25.2 |
| rs12253253AB | *CYP2C19* | 19.5 |
| rs12773342AB | *CYP2C19* | 19.1 |
| rs2756104BB | *ABCC2* | 18.0 |
| rs9341244AB | *CYP1B1* | 17.0 |
| rs12904742AB | *CYP1A2* | 15.4 |
| rs2104161BB | *CYP2C19* | 15.0 |
| rs28365067BB | *CYP3A5* | 14.4 |
| rs4086116AB | *CYP2C9* | 14.2 |
| rs9341263AB | *CYP1B1* | 13.5 |
| rs12253253BB | *CYP2C19* | 12.6 |
| rs4617515BB | *CYP2C19* | 12.4 |
| rs2070673BB | *CYP2E1* | 11.3 |
| rs6956344AB | *CYP3A4* | 10.8 |
| rs2070673AB | *CYP2E1* | 10.1 |
| rs8031941AB | *CYP1A1* | 9.8 |
| rs3740067BB | *ABCC2* | 9.3 |
| rs1934963AB | *CYP2C9* | 8.9 |
| rs2104161AB | *CYP2C19* | 8.9 |
| rs2235048AB | *ABCB1* | 8.8 |
| rs9332104BB | *CYP2C9* | 8.2 |
| rs11597626AB | *CYP2C19* | 8.1 |
| rs12268020AB | *CYP2C19* | 8.1 |
| rs9332172AB | *CYP2C9* | 7.9 |
| rs12768009AB | *CYP2C19* | 7.6 |
| rs12268020BB | *CYP2C19* | 7.5 |
| rs17216317AB | *ABCC2* | 7.1 |
| rs11597626BB | *CYP2C19* | 6.5 |
| rs2404955AB | *CYP3A4* | 6.5 |
| rs9332168AB | *CYP2C9* | 6.3 |
| rs1934963BB | *CYP2C9* | 6.2 |
| rs3740066BB | *ABCC2* | 6.0 |
| rs2070677BB | *CYP2E1* | 6.0 |
| rs2235048BB | *ABCB1* | 5.8 |
| rs11188092AB | *CYP2C19* | 5.8 |
| rs2153628BB | *CYP2C9* | 5.8 |
| rs2405184AB | *CYP3A4* | 5.6 |
| rs2153628AB | *CYP2C9* | 5.4 |
| rs4617515AB | *CYP2C19* | 5.3 |
| rs8192772AB | *CYP2E1* | 5.3 |
| rs776746AB | *CYP3A5* | 5.3 |
| rs1045642AB | *ABCB1* | 5.2 |
| rs28371764AB | *CYP3A5* | 5.0 |
| rs2515642AB | *CYP2E1* | 5.0 |
| rs9332104AB | *CYP2C9* | 4.9 |
| rs2515644AB | *CYP2E1* | 4.9 |
| rs776746BB | *CYP3A5* | 4.7 |
| rs9332168BB | *CYP2C9* | 4.6 |
| rs915907AB | *CYP2E1* | 4.6 |
| rs2515644BB | *CYP2E1* | 4.4 |
| rs11188092BB | *CYP2C19* | 4.4 |
| rs2515642BB | *CYP2E1* | 4.0 |
| Age group 3 | *-* | 3.9 |
| rs6413419AB | *CYP2E1* | 3.7 |
| rs2070677AB | *CYP2E1* | 3.5 |
| rs915906AB | *CYP2E1* | 3.4 |
| rs11568732AB | *CYP2C19* | 3.2 |
| rs28365067AB | *CYP3A5* | 3.1 |
| rs2515641AB | *CYP2E1* | 2.9 |
| rs3758580AB | *CYP2C19* | 2.6 |
| rs12768009BB | *CYP2C19* | 2.5 |
| rs2069526AB | *CYP1A2* | 2.2 |
| rs12778026AB | *CYP2C19* | 2.1 |
| rs8192772BB | *CYP2E1* | 2.0 |
| rs2253635BB | *CYP2C9* | 2.0 |
| rs162562AB | *CYP1B1* | 1.9 |
| rs28360521AB | *CYP2D6* | 1.8 |
| rs2515641BB | *CYP2E1* | 1.7 |
| rs7092584AB | *CYP2E1* | 1.6 |
| rs1137968BB | *ABCC2* | 1.4 |
| rs12773342BB | *CYP2C19* | 1.0 |
| rs4986893AB | *CYP2C19* | 1.0 |
| rs743535BB | *CYP2E1* | 1.0 |
| rs3740065BB | *ABCC2* | 1.0 |
| rs8192775BB | *CYP2E1* | 1.0 |
| rs162562BB | *CYP1B1* | 0.9 |
| rs3740066AB | *ABCC2* | 0.8 |
| rs1128503AB | *ABCB1* | 0.8 |
| rs743535AB | *CYP2E1* | 0.5 |
| rs2273697BB | *ABCC2* | 0.4 |
| rs2253635AB | *CYP2C9* | 0.2 |
| rs4304697AB | *CYP2C19* | 0.1 |

HS, hippocampal sclerosis; MDA, mean decrease accuracy
